# Editorial: Microorganisms and their metabolism affecting quality, safety and functionality of agricultural products

**DOI:** 10.3389/fmicb.2023.1215112

**Published:** 2023-05-17

**Authors:** Makoto Kimura, Hiromi Nishida, Masashi Kato, Masatoshi Goto, Tomoyuki Nakagawa

**Affiliations:** ^1^Department of Applied Biosciences, Graduate School of Bioagricultural Sciences, Nagoya University, Nagoya, Aichi, Japan; ^2^Department of Food and Life Sciences, Toyo University, Itakura, Gunma, Japan; ^3^Faculty of Agriculture, Meijo University, Nagoya, Aichi, Japan; ^4^Department of Applied Biochemistry and Food Science, Faculty of Agriculture, Saga University, Saga, Japan; ^5^Faculty of Applied Biological Sciences, Gifu University, Gifu, Japan

**Keywords:** *Aspergillus oryzae* (the *koji* mold), fermented foods and beverages, *Fusarium graminearum*, microbiome analysis, mycotoxin biosynthesis, polyunsaturated fatty acids (PUFAs), trichothecene gene regulation, yeasts

Microorganisms are closely associated with agriculture and food industries in both beneficial and detrimental ways. As the beneficial use of microorganisms, humans have taken advantage of their activities for preserving, flavoring, and enriching the agricultural products through fermentation. Since the early ages of crop cultivation, yeasts and molds inhabiting raw materials in the environment have been used for processing, which has led to the emergence of indigenous food cultures with unique tastes and odors. Despite the long history of traditional fermentation, it was not until the mid-19th century, when Louis Pasteur elucidated the chemical processes associated with life and structural integrity of yeast cells, that alcoholic fermentation has come to be recognized as the result of microbial activity (Barnett, 2003). In contrast to the positive impact of fermentation, the metabolic activities of some microorganisms can adversely affect human health by poisoning agricultural products. The description of such a negative impact was made by Louis René Tulasne in 1853, belonging to the same era as when Pasteur established the concept of fermentation, who showed that toxic ergot sclerotia were produced by a fungus, but not by the rye itself (Haarmann et al., 2009). The pathogenic *Claviceps* fungus was later shown to synthesize ergot alkaloid mycotoxins in the sclerotia, after which time the control of mycotoxins in agricultural products has become an important issue for ensuring food safety. In the light of the importance of microbial metabolism in such double-edged ways, the present Research Topic accepted five original research papers and one perspective paper (from a total of 13 submissions), which have expanded the scientific knowledge of the relevant microorganisms by using advanced modern technology, as outlined below.

*Sake* is a traditional Japanese rice wine made by advanced parallel double fermentation that involves starch saccharification of steamed rice grains by *Aspergillus oryzae* (known as the *koji* mold; the solid rice culture is called *koji*) and simultaneous alcoholic fermentation by the yeast *Saccharomyces cerevisiae*. Other than the common eukaryotic fermenting microorganisms used in *sake* production, bacteria inhabiting the *sake* breweries (described as *kuratsuki* in Japanese) have been identified by using massive parallel DNA sequencing, revealing the diversity of microbial flora during *sake* production in different breweries. This finding has led to the hypothesis that the specific flavor and taste of *sake* products possibly arose from interactions between brewery-specific *kuratsuki* bacteria and yeasts (Nishida, 2021). Indeed, functional studies of such recently isolated and characterized *kuratsuki* bacteria have provided insights into their involvement in making the flavor and taste of *sake* during the brewing process (Yazaki and Nishida, 2023). In close connection with this theory, Ito et al. describe the diversity and dynamics of the microbiome during the production process of *sake* at a local brewery in Kawaba village, a relatively isolated mountainous area bordering north of the Kanto Plain in Japan. By comparing the amplicon sequence variants of the 16S rRNA gene in the fermentation starter *moto* vs. the external environment, they demonstrated that autochthonous bacteria in the built environments, equipment surfaces, and raw materials have moved into *moto* in the brewery. It is also noteworthy that the relative abundances of microbes from the external environment have changed during *moto* fermentation.

In addition to the metagenomics studies of traditional *sake* manufacturing process, this Research Topic includes two papers, in which the authors sought to improve fermentation and microbial products for industrial applications. Liu et al. describe their efforts to improve the quality of cider, a widely consumed alcoholic beverage produced by the fermentation of apple juice. It has been known that wild microbes present in the pericarps and stems often interfere with natural fermentation by autochthonous yeasts, thereby preventing high-quality cider production on an industrial scale (Lorenzini et al., 2019). With the ultimate goal of improving the quality and style of ciders, they screened and selected yeast starter strains with diverse characteristics by using a conventional olfactory test. Several candidate strains were characterized in terms of sugar utilization, methanol production, and volatile profiles, which could be explained by the relative transcription levels of genes associated with sugar metabolism, stress tolerance, and/or aroma production. The application of new strains that complement the conventional natural fermentation by autochthonous yeasts is expected to satisfy consumer demand and promote cider industry development. Another study by Wang et al. deals with polyunsaturated fatty acids (PUFAs), such as eicosapentaenoic acid (EPA) and docosahexaenoic acid (DHA), which have numerous health benefits throughout human life. For the production of PUFAs, *Schizochytrium* is considered as one of the alternative sources to fish oils, as this eukaryotic heterotrophic microalagae can produce high levels of such functional food ingredients (Chang et al., 2013). As a first step toward achieving an industrial-level of PUFA production by *Schizochytrium*, the authors explored the possibility of using chemical modulators. Consequently, they found an increased ratio of PUFAs to saturated fatty acids, as well as increased production of PUFAs, by the addition of 1,3-propanediol. In cultures of *Schizochytrium* amended with varying doses and ratios of propanol and 1,3-propanediol, total fatty acid synthesis was synergistically enhanced at optimized concentrations.

Apart from the applied aspects of microorganisms used for the production of fermented foods and beverages, two basic studies on the cell structure and gene functions of *A. oryzae* have expanded the knowledge of this important food-fermentation fungus. One problem that hampers the breeding of the *koji* mold is its inability to cross genetically, as sexual reproduction has not yet been discovered. To enhance its potential applications in the industry, it is important to develop breeding techniques and increase its genetic diversity so that strains with beneficial traits can be selected. Toward this end, Katayama and Maruyama investigated the regulatory mechanisms of sclerotia formation, because this asexual structure serves as a repository for sexual development in closely related *Aspergillus flavus* (Kwon-Chung and Sugui, 2009). They showed inhibition of sclerotia formation and conidia induction by trace amounts of copper, and elucidated that the mechanism is mediated via transcriptional activation of *AobrlA*, a critical transcription factor gene for conidiation. Molecular genetic analyses have established epistatic relationships among the genes encoding AoSod1 (copper-dependent superoxide dismutase), AoCcsA (a copper chaperone that introduces copper ions into AoSod1), and EcdR (a transcription factor for *AobrlA* expression) in the copper regulation of these reproductive structures. In addition to the molecular mechanism underlying sterility, the presumed occurrence of galactofuranose (Gal*f* )-containing glycans in *A. oryzae* cell walls (Nakajima and Ichishima, 1994) led to investigations by Kadooka et al., who identified the related UDP-galactofuranose mutase gene (*ugmA*) and showed the UgmA-dependent synthesis of Gal*f*-containing glycoproteins in the *koji* mold. Although the amount of the Gal*f*-containing glycoproteins in the cell wall fraction was significantly smaller than that in other *Aspergillus* species, the incorporation of Gal*f* side chains into the core mannan backbone was required for the maintenance of cell wall integrity; i.e., as revealed by the temperature and calcofluor white sensitivities of the *A. oryzae* Δ*ugmA* disruptant. Given that such fungal-type galactomannan (FTGM) is a remnant of the ancestral pathogenic *Aspergillus* species and is antigenic in humans, it is intriguing to assume that some health-promoting benefits, such as immunostimulatory effects and intestinal eubiosis, occur through habitual ingestion of *koji*-fermented foods (Kitagaki, 2021). These findings provide a theoretical basis for innovative industrial developments.

*Fusarium graminearum* and other closely related *Fusarium* phytopathogenic fungi infect wheat and barley, and accumulate trichothecene mycotoxins in the grains (Kimura et al., 2007). This group of mycotoxins inhibits protein translation in eukaryotes and causes moldy-grain toxicoses through ingestion of contaminated agricultural products. These *Fusarium* species have recently been found to have a potential ability to glucosylate the side chains of the trichothecene skeleton, raising the possibility of producing diverse variations of “masked” mycotoxins (Matsui et al., 2021). Thus, the mechanism of regulation of trichothecene biosynthesis by *F. graminearum* has been the subject of intensive studies, and several hypotheses and models have been proposed. Liew et al. discuss several issues that previous researchers had not noticed, presenting original unpublished results, and proposing a revision of the current understanding of the mechanism of trichothecene biosynthesis regulation. Finally, the perspective paper shares a strategy for establishing a regulatory model of transcription of *Tri6* and *Tri10* in *F. graminearum*, which are trichothecene transcription factor and regulatory protein genes, respectively, located at the core region of the gene cluster (Maeda et al., 2018). Overall, the information is highly suggestive for research on the roles of various genetic and environmental factors involved in the regulation of secondary metabolism.

In summary, this Research Topic highlights recent advances that explore applied aspects of microorganisms for the production of fermented foods and beverages, as well as basic research into the genetic and biochemical mechanisms underlying the growth, development, and metabolism of microorganisms that affect the quality, safety, and functionality of agricultural products.

## Author contributions

All authors listed have made a substantial, direct, and intellectual contribution to the work and approved it for publication.
